# Neoadjuvant Treatment and Postoperative Complications After Surgery for Esophageal Cancer: A Population-Based, Nationwide Study in Finland

**DOI:** 10.1245/s10434-025-17945-y

**Published:** 2025-08-06

**Authors:** Ville E. J. Sirviö, Jari V. Räsänen, Olli Helminen, Mika Helmiö, Heikki Huhta, Raija Kallio, Vesa Koivukangas, Arto Kokkola, Elina Lietzen, Sanna Meriläinen, Vesa-Matti Pohjanen, Tuomo Rantanen, Ari Ristimäki, Juha Saarnio, Eero Sihvo, Tuula Tyrväinen, Mikko Uimonen, Antti Valtola, Joonas H. Kauppila

**Affiliations:** 1 Department of Oesophageal and General Thoracic Surgery, Helsinki University Hospital and University of Helsinki, Helsinki, Finland; 2 Surgery Research Unit, Oulu University Hospital and University of Oulu, Oulu, Finland; 3 The Division of Digestive Surgery and Urology, Turku University Hospital and University of Turku, Turku, Finland; 4 Department of Oncology and Radiotherapy, Oulu University Hospital, Oulu, Finland; 5 Department of Surgery, University of Helsinki and Helsinki University Hospital, Helsinki, Finland; 6 Cancer and Translational Medicine Research Unit, Medical Research Center Oulu, University of Oulu and Oulu University Hospital, Oulu, Finland; 7 Department of Surgery, University of Eastern Finland and Kuopio University Hospital, Kuopio, Finland; 8 Department of Pathology and HUS Diagnostic Center, University of Helsinki and Helsinki University Hospital, Helsinki, Finland; 9 Applied Tumor Genomics Research Program, Research Programs Unit, University of Helsinki, Helsinki, Finland; 10 Department of Surgery, Central Finland Central Hospital, Jyväskylä, Finland; 11 Department of Gastroenterology and Alimentary Tract Surgery, Tampere University Hospital, Tampere, Finland; 12 Heart Hospital, Tampere University Hospital, Wellbeing Services County of Pirkanmaa, Tampere, Finland; 13 Upper Gastrointestinal Surgery, Department of Molecular Medicine and Surgery, Karolinska Institutet, Stockholm, Sweden

**Keywords:** Esophageal cancer, Esophagectomy, Surgery, Surgical complications, Neoadjuvant treatment, Chemotherapy, Chemoradiotherapy

## Abstract

**Background:**

The role of neoadjuvant treatment and its modalities in the risk of complications after esophagectomy in national practice is unclear. The aim of this study was to compare postoperative complications after neoadjuvant treatment (nT) and upfront surgery (US), neoadjuvant chemoradiotherapy (nCRT) and neoadjuvant chemotherapy (nCT), and to assess the effect of complications from neoadjuvant treatment in surgical risk.

**Patients and Methods:**

All patients undergoing esophagectomy for esophageal cancer in Finland in 2005–2016 were included. Incidences of all postoperative complications defined by the Esophagectomy Complications Consensus Group as well as major complications, 90-day mortality, and hospital and ICU stay were reported stratified by treatment strategy (nT versus US, nCRT versus nCT, nT-complication versus no complication). Primary outcomes were compared using logistic regression in these patient groups.

**Results:**

Out of 774 patients, 423 (55%) had nT and 351 (45%) had US. Of the 423 patients undergoing nT, 249 (59%) had nCT, 3 (1%) had radiotherapy only, and 171 (40%) had nCRT. After adjusting for relevant confounders, there were no increases in pneumonia, anastomotic leak, major complications, overall complications, or 90-day mortality with nT compared with US, nCRT compared with nCT, or in patients with nT complications compared with those without.

**Conclusions:**

This nationwide, population-based study reports no difference in postoperative complications or mortality after nT compared with US or nCRT compared with nCT. Patients with neoadjuvant treatment complications were not at higher risk of postoperative complications.

**Supplementary Information:**

The online version contains supplementary material available at 10.1245/s10434-025-17945-y.

Esophageal cancer is the sixth most common cause of cancer death worldwide.^1^ Curative-intent treatment of locally advanced esophageal cancer consists of neoadjuvant treatment followed by esophagectomy, associated with a high risk of surgical complications and postoperative morbidity.^2^

Long-term survival benefits for both neoadjuvant chemotherapy (nCT) and neoadjuvant chemoradiotherapy (nCRT) have been well established.^3–5^ In some randomized studies, nCRT has been associated with increased short-term mortality or morbidity compared with surgery alone, which has not been seen with nCT.^6–8^ For patients undergoing neoadjuvant treatment (nT), current treatment guidelines^2,9^ recommend perioperative chemotherapy or the carboplatin, paclitaxel, radiotherapy (CROSS) protocol for locally advanced disease, however, evidence supporting the superiority of either approach in terms of operative safety has been scarce. Randomized studies^10,11^ as well as the only existing larger observational studies have had contradicting results on the matter.^12,13^ The role of nT and its modalities in surgical risk, therefore, remains unclear in daily clinical practice.

Another concern in surgical safety is toxic complications from chemo(radio)therapy, due to which patients might be considered as of higher operative risk, or even have surgery withheld as a treatment option. There are currently no larger studies investigating differences in postoperative complications in patients forced to abort neoadjuvant treatment or proceed treatment with a reduced dosage, compared with patients tolerating full intended neoadjuvant treatment.

Further nationwide reports using standardized outcome definitions are needed to determine the risks of the treatment provided to patients in everyday care, and to allow for international comparison in an effort to maximize patient safety.

The aim of this population-based, nationwide study was to assess the incidence of surgical complications in patients undergoing nT compared with those undergoing US, and in patients undergoing nCRT compared with those undergoing nCT. A secondary aim was to compare surgical complications after esophagectomy between patients with aborted or dose-reduced neoadjuvant treatment (A/R) and patients receiving full intended neoadjuvant treatment.

## Patients and Methods

### Study Design

This study was a nationwide Finnish, population-based, retrospective cohort study.

### Data Sources

The Finnish National Esophago-Gastric Cancer Cohort (FINEGO), including all patients undergoing esophagectomy for esophageal cancer in Finland during the years 1987–2016, was used as the data source for this study.^14^ Patient identification for the cohort was carried out by searching for diagnosis and operation codes from previously validated and complete national registries.^15–17^ Identified patients were screened by expert surgeons before final inclusion. Detailed clinical variables such as comorbidity, tumor histology, tumor stage, neoadjuvant treatment status, surgical technique, anastomosis location, and resection radicality were retrieved from patient records by expert thoracic and upper-gastrointestinal surgeons, and basic patient information such as age, sex, and year of diagnosis were provided by the national registries. Surgical complications were retrieved and classified by the Esophagectomy Complications Consensus Group (ECCG) criteria^18^ and graded for severity according to the Clavien–Dindo (CD) classification.^19^

### Inclusion Criteria

Patients in the FINEGO-database identified through either the Finnish Cancer Registry or the Finnish Patient Registry and undergoing esophagectomy for esophageal cancer during the years 2005–2016 in Finland were included.

### Exposure

The primary exposures were neoadjuvant treatment compared with upfront surgery and neoadjuvant chemoradiotherapy compared with neoadjuvant chemotherapy. The secondary exposure was aborted or dose-reduced neoadjuvant treatment compared with full intended neoadjuvant treatment.

### Outcomes

Outcomes were recorded for 90 days after esophagectomy. The primary outcomes were surgical complications defined by ECCG, major complications, pneumonia, anastomotic leakage, and 90-day mortality. Secondary outcomes were complication categories defined by ECCG, length of ICU stay, and length of hospital stay.

Complications and complication categories were defined as per the ECCG framework. Major complications were defined as CD IIIa or higher. Reoperations were defined as surgical interventions done in the OR with or without general anesthesia, excluding endoscopic interventions; 90-day mortality was defined as all-cause mortality during 90 days from surgery.

### Statistical Analysis

Patient and tumor characteristics were presented as frequencies and percentages stratified by treatment strategy. Differences in patient and tumor characteristics between treatment groups were assessed using chi-squared test and *t*-test.

Postoperative outcomes, entailing all unique complications defined in the ECCG framework, complication categories defined by ECCG, major complications, reoperations, lengths of ICU and hospital stay, and 90-day mortality were presented as frequencies and percentages. Outcomes were stratified by treatment strategy (nT versus US), neoadjuvant treatment modality (nCRT versus nCT), and finally by the completement status of neoadjuvant treatment (A/R versus full).

Logistic regression provided ORs with 95% confidence intervals (CI), and survival outcomes were analyzed with Cox regression providing HRs with 95% CI. The first model was a crude model not adjusted for other variables. The main model was adjusted for relevant confounders: year of surgery (continuous), age (continuous), sex (male or female), tumor stage (0–I, II, III, or IV), comorbidity (CCI 0, 1, or 2 or more), histology (adenocarcinoma or squamous cell carcinoma), and surgical strategy (minimally invasive or open resection).

Firstly, the outcomes in nT were compared with US. Secondly, an analysis including patients receiving neoadjuvant treatment was conducted, comparing nCRT with nCT. Lastly, patients receiving neoadjuvant treatment were included, and those with aborted or dose-reduced neoadjuvant treatment were compared with those undergoing full intended treatment.

## Results

A total of 774 patients undergoing esophagectomy for esophageal cancer in Finland from 1 January 2005 to 31 December 2016 were identified in national registries for inclusion in the FINEGO database. Of these, 423 (55%) had preoperative neoadjuvant treatment and 351 (45%) had upfront surgery. Of all patients, 78% were men, median age was 64 years, and most patients had no comorbidities (54%). The distributions of patient sex, CCI, histology, and tumor location were similar between groups, while there were statistically significant differences in age, clinical and pathological stage, mode of surgery, and lymph node yield. The preferred surgical approach was Ivor–Lewis esophagectomy, however, there were significantly more transhiatal procedures in the US group. Minimally invasive esophagectomies were performed in 38% of patients in the nT group and 18% in the US group (Table 1).

**Table 1:** Characteristics of patients undergoing neoadjuvant treatment and upfront surgery in Finland in 2005–2016

	nT	US	Total	
	*n* (%)	*n* (%)	*n* (%)	*p*-Value
Total	423 (100.0)	351 (100.0)	774 (100.0)	
*Sex*				0.220
Male	335 (79.2)	265 (75.5)	600 (77.5)	
Female	88 (20.8)	86 (24.5%)	174 (22.5)	
*Age*				**< 0.001**
Median (IQR)	63 (57–69)	66 (59–72)	64 (58–71)	
*Clinical stage*				**< 0.001**
0	0 (0.0)	17 (4.9)	17 (2.2)	
I	1 (0.2)	83 (23.9)	84 (10.9)	
II	46 (10.9)	96 (27.7)	142 (18.4)	
III	297 (70.2)	136 (39.2)	433 (56.2)	
IV	79 (18.7)	15 (4.3)	94 (12.2)	
*Pathological stage*				**< 0.001**
0	37 (8.9)	11 (3.1)	48 (6.3)	
I	87 (20.9)	125 (35.7)	212 (27.6)	
II	76 (18.2)	54 (15.4)	130 (16.9)	
III	171 (41.0)	124 (35.4)	295 (38.5)	
IV	46 (11.0)	36 (10.3)	82 (10.7)	
*CCI*				0.380
0	232 (54.8)	184 (52.4)	416 (53.7)	
1	128 (30.3)	100 (28.5)	228 (29.5)	
2	40 (9.5)	47 (13.4)	87 (11.2)	
≥ 3	23 (5.4)	20 (5.7)	43 (5.6)	
*Histology*				0.447
Adenocarcinoma	321 (75.9)	258 (73.5)	579 (74.8)	
Squamous cell carcinoma	102 (24.1)	93 (26.5)	195 (25.2)	
*Tumor location*				0.539
Upper	8 (1.9)	8 (2.3)	16 (2.1)	
Middle	56 (13.2)	51 (14.5)	107 (13.8)	
Lower	208 (49.2)	184 (52.4)	392 (50.6)	
GO-junction	151 (35.7)	108 (30.8)	259 (33.5)	
*Mode of surgery*				**< 0.001**
Open	223 (52.7)	275 (78.3)	498 (64.3)	
Hybrid	40 (9.5)	14 (4.0)	54 (7.0)	
tMIO	160 (37.8)	62 (17.7)	222 (28.7)	
*Type of resection*				**< 0.001**
Ivor–Lewis	318 (75.2)	183 (52.1)	501 (64.7)	
McKeown	81 (19.1)	64 (18.2)	145 (18.7)	
Transhiatal	14 (3.3)	93 (26.5)	107 (13.8)	
Left thoracoabdominal	1 (0.2)	1 (0.3)	2 (0.3)	
Proximal gastrectomy	1 (0.2)	2 (0.6)	3 (0.4)	
Combined gastro-esophagectomy	8 (1.9)	8 (2.3)	16 (2.1)	
*Conduit material*				**0.011**
Stomach	413 (97.6)	326 (92.9)	739 (95.5)	
Small intestine	3 (0.7)	10 (2.8)	13 (1.7)	
Colon	6 (1.4)	10 (2.8)	16 (2.1)	
Unclear or other	1 (0.2)	5 (1.4)	6 (0.8)	
*Location of anastomosis*				**< 0.001**
Neck	91 (21.5)	147 (41.9)	238 (30.7)	
Thorax	331 (78.3)	202 (57.5)	533 (68.9)	
Unclear or no anastomosis	1 (0.2)	2 (0.6)	3 (0.4)	
*Resection radicality*				0.152
R0	383 (91.0)	310 (88.6)	23 (3.0)	
R1	30 (7.1)	25 (7.1)	55 (7.1)	
R2	8 (1.9)	15 (4.3)	693 (89.9)	
*Lymph node yield*				**< 0.001**
0–16	174 (41.6)	200 (57.8)	374 (49.0)	
≥ 17	244 (58.4)	146 (42.2)	390 (51.0)	
*Neoadjuvant type*				
nCT	249 (58.9)	–	–	
Radiotherapy	3 (0.7)	–	–	
nCRT	171 (40.4)	–	–	
*Neoadjuvant protocol*				
EOX or derivative	232 (55.2)	–	–	
TP (other than cross)	38 (9.0)	–	–	
CROSS	39 (9.3)	–	–	
PF/XELOX	69 (16.4)	–	–	
TPF	9 (2.1)	–	–	
Single agent	29 (6.9)	–	–	
Unknown	4 (1.0)	–	–	
*Completion of neoadjuvant treatment*				
Full	305 (72.6)	–	–	
A/R	96 (22.7)	–	–	
Missing	19 (4.5)	–	–	

Statistical significance indicated by bold font

*nT* Neoadjuvant treatment, *US* Upfront surgery, *IQR* Interquartile range, *CCI* Charlson comorbidity index, *tMIO* Totally minimally invasive esophagectomy, *nCT* Neoadjuvant chemotherapy, *nCRT* Neoadjuvant chemoradiotherapy, *CROSS* Carboplatin-paclitaxel and radiotherapy, *EOX* Epirubicin-platinum-capecitabine/fluorouracil, *PF/XELOX* Platinum-fluorouracil/capecitabine, *TP* Taxane-platinum, *TPF* Taxane-platinum-fluorouracil/capecitabine, *A/R* Aborted or dose-reduced neoadjuvant treatment

Of the 423 patients undergoing nT, 249 (59%) had nCT, 3 (1%) had radiotherapy only, and 171 (40%) had nCRT. EOX-derived therapies using three agents (EOX, EOF, ECX or ECF, i.e., epirubicin-oxali-/carboplatinum-capecitabine/fluorouracil) were the most used protocols at 55% of patients, followed by 16% of patients with platinum-fluorouracil/capecitabine (with PF/XELOX), 9% with carboplatin-paclitaxel and radiotherapy (CROSS), and 9% with taxane-platinum (TP). Neoadjuvant treatment complications resulting in treatment discontinuation or dose-reduction occurred in 96 (23%) of patients (Table 1).

### Neoadjuvant Treatment versus Upfront Surgery

Major complications occurred in 41.8% of patients undergoing nT and in 48.1% of those undergoing US; 18.7% of patients undergoing nT and 19.1% of patients undergoing US underwent reoperations. The 90-day mortality rate was slightly higher in patients undergoing US (4.0% nT versus 6.6% US). Lengths of ICU and hospital stay were similar (Table 2).

**Table 2:** The 90-day outcomes of patients undergoing neoadjuvant treatment and upfront surgery for esophageal cancer

	nT	US
	*n* (%)	*n* (%)
Patients	423 (100.0)	351 (100.0)
Complications defined by ECCG		
*Pulmonary*	*170 (40.2)*	*156 (44.4)*
Pneumonia	90 (21.3)	76 (21.7)
Pleural effusion requiring additional drainage procedure	91 (21.5)	85 (24.2)
Pneumothorax requiring treatment	12 (2.8)	16 (4.6)
Atelectasis mucous plugging requiring bronchoscopy	32 (7.6)	16 (4.6)
Respiratory failure requiring reintubation	28 (6.6)	36 (10.3)
Acute aspiration	17 (4.0)	9 (2.6)
Acute respiratory distress syndrome	13 (3.1)	10 (2.8)
Tracheobronchial injury	2 (0.5)	0 (0.0)
Chest tube maintenance for air leak for > 10 days postoperatively	3 (0.7)	3 (0.9)
*Cardiac*	*70 (16.5)*	*61 (17.4)*
Cardiac arrest requiring CPR	2 (0.5)	7 (2.0)
Myocardial infarction	0 (0.0)	3 (0.9)
Dysrhythmia atrial requiring treatment	65 (15.4)	52 (14.8)
Dysrhythmia ventricular requiring treatment	1 (0.2)	1 (0.3)
Congestive heart failure requiring treatment	6 (1.4)	8 (2.3)
Pericarditis requiring treatment	0 (0.0)	0 (0.0)
*Gastrointestinal*	*98 (23.2)*	*75 (21.4)*
Anastomotic leak	47 (11.1)	35 (12.3)
Type 1	5 (1.2)	8 (2.3)
Type 2	25 (5.9)	15 (4.3)
Type 3	17 (4.0)	20 (5.7)
Conduit necrosis	16 (3.8)	11 (3.1)
Type 1	6 (1.4)	3 (0.9)
Type 2	1 (0.2)	0 (0.0)
Type 3	9 (2.1)	8 (2.3)
Ileus defined as small bowel dysfunction preventing or delaying enteral feeding	8 (1.9)	4 (1.1)
Small bowel obstruction	2 (0.5)	0 (0.0)
Feeding J-tube complication	12 (2.8)	8 (2.3)
Pyloromyotomy/pyloroplasty complication	4 (0.9)	4 (1.1)
*Clostridium difficile* infection	4 (0.9)	4 (1.1)
Gastrointestinal bleeding requiring intervention or transfusion	5 (1.2)	6 (1.7)
Delayed conduit emptying requiring intervention or delaying discharge or requiring maintenance of NG drainage > 7 days postoperatively	5 (1.2)	8 (2.3)
Pancreatitis	1 (0.2)	0 (0.0)
Pancreatic fistula	2 (0.5)	0 (0.0)
Liver dysfunction	0 (0.0)	1 (0.3)
Biliary leakage	2 (0.5)	0 (0.0)
*Urologic*	*14 (3.3)*	*11 (3.1)*
Acute renal insufficiency (defined as doubling of baseline creatinine)	11 (2.6)	6 (1.7)
Acute renal failure requiring dialysis	2 (0.5)	1 (0.3)
Urinary tract infection	3 (0.7)	2 (0.6)
Urinary retention requiring reinsertion of urinary catheter, delaying discharge, or discharge with urinary catheter	0 (0.0)	3 (0.9)
*Thromboembolic*	*16 (3.8)*	*14 (4.0)*
Deep venous thrombosis	1 (0.2)	1 (0.3)
Pulmonary embolus	14 (3.3)	11 (3.1)
Stroke (CVA)	1 (0.2)	3 (0.9)
Peripheral thrombophlebitis	1 (0.2)	0 (0.0)
*Neurologic*	*47 (11.1)*	*53 (15.1)*
Recurrent nerve injury	17 (4.0)	31 (8.8)
Type 1	16 (3.8)	30 (8.5)
Type 2	1 (0.2)	0 (0.0)
Type 3	0 (0.0)	1 (0.3)
Other neurologic injury	6 (1.4)	9 (2.6)
Acute delirium	25 (5.9)	13 (3.7)
Delirium due to alcohol withdrawal	1 (0.2)	2 (0.6)
*Infectious*	*65 (15.4)*	*67 (19.1)*
Wound infection requiring opening wound or antibiotics	13 (3.1)	21 (6.0)
Central IV-line infection requiring removal or antibiotics	5 (1.2)	6 (1.7)
Intraabdominal abscess	7 (1.7)	7 (2.0)
Intrathoracic abscess	24 (5.7)	21 (6.0)
Sepsis defined as positive blood culture	15 (3.5)	17 (4.8)
Other infections requiring antibiotics	21 (5.0)	16 (4.6)
*Wound/diaphragm*	*13 (3.1)*	*12 (3.4)*
Thoracic wound dehiscence	3 (0.7)	4 (1.1)
Abdominal wall wound dehiscence	10 (2.4)	7 (2.0)
Acute abdominal wall hernia	0 (0.0)	3 (0.9)
Acute diaphragmatic hernia	0 (0.0)	0 (0.0)
*Other*	*74 (17.5)*	*58 (16.5)*
Chyle leak		
Type 1	2 (0.5)	3 (0.9)
Type 2	16 (3.8)	2 (0.6)
Type 3	9 (2.1)	10 (2.8)
Reoperation for other reasons than bleeding, anastomotic leak, or conduit necrosis	51 (12.1)	43 (12.3)
Multiple organ dysfunction syndrome	3 (0.7)	4 (1.1)
Major complications	177 (41.8)	169 (48.1)
Reoperations	79 (18.7)	67 (19.1)
ICU stay (IQR)	2 (1–4)	2 (1–4)
Hospital stay (IQR)	14 (11.75–21)	15 (12–22.25)
90-day mortality	17 (4.0)	23 (6.6)
Patients receiving adjuvant therapy		
Not planned	171 (40.4)	206 (58.9)
Planned but not started	7 (1.7)	5 (1.4)
Planned and started, but terminated	18 (4.3)	6 (1.7)
Completed	113 (26.7)	73 (20.9)
*Planned to be done in another hospital	28 (6.6)	20 (5.7)
Unclear	86 (20.3)	40 (11.4)

^*^No follow-up information available

*nT* Neoadjuvant treatment, *US* Upfront surgery, *ECCG* Esophagectomy Complications Consensus Group, *CPR* Cardiopulmonary resuscitation, *NG* Nasogastric, *CVA* Cerebrovascular accident, *IV* Intravenous, *ICU* Intensive care unit, *IQR* Interquartile range

For specific complications, the rate of pneumonia was similar between groups (21.3% nT versus 21.7% US). Anastomotic leak occurred in 11.1% of patients undergoing nT and in 12.3% of patients undergoing US, with slightly more type III leakage needing surgical intervention in the US group. The rate of conduit necrosis was similar (3.8% nT versus 3.1% US). Recurrent nerve injury was more common in the US group at 8.8% compared with 4.0% in the nT group. No significant differences were seen in the rates of other specific complications (Table 2).

Incidence rates were similar in cardiac, gastrointestinal, urologic, thromboembolic, wound/diaphragm, and other complications. Minor differences were seen in pulmonary (40.2% nT versus 44.4% US), neurologic (11.1% nT versus 15.1% US), and infectious complications (15.4% nT versus 19.1% US) (Table 2).

In regression analyses, nT was associated with a higher risk of overall complications [OR 0.72 (0.53–0.97)] in the crude model. After adjusting for confounding, the difference was attenuated [OR 0.86 (0.61–1.21)]. Neoadjuvant treatment was not associated with higher risk of overall complications, major complications, pneumonia, anastomotic leak, 90-day mortality, or any of the complication categories (Table 5).

### Neoadjuvant Chemoradiotherapy versus Neoadjuvant Chemotherapy

Major complications occurred in 44.4% of patients undergoing nCRT and 40.2% of patients undergoing nCT. Reoperation rates were similar at 19.6% for nCRT and 18.5% for nCT; 90-day mortality was lower in patients undergoing nCRT (2.9% nCRT versus 4.8% nCT). Lengths of ICU and hospital stay were similar (Table 3).

**Table 3:** The 90-day outcomes of patients undergoing neoadjuvant chemoradiotherapy and neoadjuvant chemotherapy prior to esophagectomy for esophageal cancer

	nCRT	nCT
	*n* (%)	*n* (%)
Patients	171 (100.0)	249 (100.0)
Complications defined by ECCG		
*Pulmonary*	*74* (*43.3*)	*95* (*38.2*)
Pneumonia	47 (27.5)	43 (17.3)
Pleural effusion requiring additional drainage procedure	37 (21.6)	53 (21.3)
Pneumothorax requiring treatment	6 (3.5)	6 (2.4)
Atelectasis mucous plugging requiring bronchoscopy	18 (10.5)	14 (5.6)
Respiratory failure requiring reintubation	16 (9.4)	12 (4.8)
Acute aspiration	9 (5.3)	8 (3.2)
Acute respiratory distress syndrome	7 (4.1)	6 (2.4)
Tracheobronchial injury	1 (0.6)	1 (0.4)
Chest tube maintenance for air leak for > 10 days postoperatively	1 (0.6)	2 (0.8)
*Cardiac*	*31* (*18.1*)	*39* (*15.7*)
Cardiac arrest requiring CPR	1 (0.6)	1 (0.4)
Myocardial infarction	0 (0.0)	0 (0.0)
Dysrhythmia atrial requiring treatment	28 (16.4)	37 (14.9)
Dysrhythmia ventricular requiring treatment	0 (0.0)	1 (0.4)
Congestive heart failure requiring treatment	3 (1.8)	3 (1.2)
Pericarditis requiring treatment	0 (0.0)	0 (0.0)
*Gastrointestinal*	*43* (*25.1*)	*54* (*21.7*)
Anastomotic leak	19 (12.6)	28 (11.2)
Type 1	3 (1.8)	2 (0.8)
Type 2	10 (5.8)	15 (6.0)
Type 3	6 (3.5)	11 (4.4)
Conduit necrosis	10 (6.6)	6 (2.4)
Type 1	4 (2.3)	2 (0.8)
Type 2	0 (0.0)	1 (0.4)
Type 3	6 (3.5)	3 (1.2)
Ileus defined as small bowel dysfunction preventing or delaying enteral feeding	4 (2.3)	4 (1.6)
Small bowel obstruction	2 (1.2)	0 (0.0)
Feeding J-tube complication	4 (2.3)	8 (3.2)
Pyloromyotomy/pyloroplasty complication	3 (1.8)	1 (0.4)
*Clostridium difficile* infection	1 (0.6)	3 (1.2)
Gastrointestinal bleeding requiring intervention or transfusion	2 (1.2)	2 (0.8)
Delayed conduit emptying requiring intervention or delaying discharge or requiring maintenance of NG drainage > 7 days postoperatively	3 (1.8)	2 (0.8)
Pancreatitis	1 (0.6)	0 (0.0)
Pancreatic fistula	0 (0.0)	2 (0.8)
Liver dysfunction	0 (0.0)	0 (0.0)
Biliary leakage	1 (0.6)	1 (0.4)
*Urologic*	*2 (1.2)*	*12 (4.8)*
Acute renal insufficiency (defined as doubling of baseline creatinine)	2 (1.2)	9 (3.6)
Acute renal failure requiring dialysis	0 (0.0)	2 (0.8)
Urinary tract infection	0 (0.0)	3 (1.2)
Urinary retention requiring reinsertion of urinary catheter, delaying discharge, or discharge with urinary catheter	0 (0.0)	0 (0.0)
*Thromboembolic*	*4* (*2.3*)	*12* (*4.8*)
Deep venous thrombosis	0 (0.0)	1 (0.4)
Pulmonary embolus	3 (1.8)	11 (4.4)
Stroke (CVA)	1 (0.6)	0 (0.0)
Peripheral thrombophlebitis	0 (0.0)	1 (0.4)
*Neurologic*	*25* (*14.6*)	*12* (*8.4*)
Recurrent nerve injury	12 (7.9)	4 (1.6)
Type 1	11 (6.4)	4 (1.6)
Type 2	1 (0.6)	0 (0.0)
Type 3	0 (0.0)	0 (0.0)
Other neurologic injury	2 (1.2)	4 (1.6)
Acute delirium	12 (7.0)	13 (5.2)
Delirium due to alcohol withdrawal	1 (0.6)	0 (0.0)
*Infectious*	*26* (*15.2*)	*39* (*15.7*)
Wound infection requiring opening wound or antibiotics	7 (4.1)	6 (2.4)
Central IV-line infection requiring removal or antibiotics	2 (1.2)	3 (1.2)
Intraabdominal abscess	4 (2.3)	3 (1.2)
Intrathoracic abscess	7 (4.1)	17 (6.8)
Sepsis defined as positive blood culture	5 (2.9)	10 (4.0)
Other infections requiring antibiotics	7 (4.1)	14 (5.6)
*Wound/diaphragm*	*6* (*3.5*)	*7* (*2.8*)
Thoracic wound dehiscence	1 (0.6)	2 (0.8)
Abdominal wall wound dehiscence	5 (2.9)	5 (2.0)
Acute abdominal wall hernia	0 (0.0)	0 (0.0)
Acute diaphragmatic hernia	0 (0.0)	0 (0.0)
*Other*	*34* (*19.9*)	*40* (*16.1*)
Chyle leak		
Type 1	1 (0.6)	1 (0.4)
Type 2	7 (4.1)	9 (3.6)
Type 3	3 (1.8)	6 (2.4)
Reoperation for other reasons than bleeding, anastomotic leak, or conduit necrosis	24 (14.0)	27 (10.8)
Multiple organ dysfunction syndrome	2 (1.2)	1 (0.4)
Major complications	76 (44.4)	100 (40.2)
Reoperations	33 (19.6)	46 (18.5)
ICU stay (IQR)	2 (1–4)	2 (1–3)
Hospital stay (IQR)	15 (12–22)	14 (11–19)
90-day mortality	5 (2.9)	12 (4.8)
Patients receiving adjuvant therapy		
Not planned	108 (63.2)	61 (24.4)
Planned but not started	1 (0.6)	6 (2.4)
Planned and started, but terminated	5 (2.9)	13 (5.2)
Completed	16 (9.4)	97 (38.8)
*Planned to be done in another hospital	8 (4.7)	20 (8.0)
Unclear	33 (19.3)	53 (21.2)

^*^No follow-up information available

*nCRT* Neoadjuvant chemoradiotherapy, *nCT* Neoadjuvant chemotherapy, *ECCG* Esophagectomy Complications Consensus Group, *CPR* Cardiopulmonary resuscitation, *NG* Nasogastric, *CVA* Cerebrovascular accident, *IV* Intravenous, *ICU* Intensive care unit, *IQR* Interquartile range

Pneumonia was more common in patients undergoing nCRT (27.5% nCRT versus 17.3% nCT). There were also more atelectasis and respiratory failures in patients undergoing nCRT. Rates of anastomotic leakage were similar, whereas conduit necrosis was more common with nCRT (6.6% nCRT versus 2.4% nCT). Patients undergoing nCRT also had more recurrent nerve injury compared with nCT (7.9% nCRT versus 1.6% nCT) (Table 3).

In complication categories, pulmonary (43.3% nCRT versus 38.2% nCT) and neurologic (14.6% nCRT) versus 8.4% nCT) complications were more common with nCRT. There were more cardiac, gastrointestinal, and other complications in the nCRT group, whereas urologic and thromboembolic complications were slightly more common with nCT. Rates of infectious and wound/diaphragm complications were similar (Table 3).

In regression analyses, nCRT was associated with a higher risk of pneumonia (OR 1.82 (1.14–2.91) and neurological complications [OR 1.86 (1.00–3.44)] in the crude model. After adjusting for confounding, these differences were attenuated [OR 1.23 (0.68–2.23) and OR 1.15 (0.52–2.56), respectively], and there were no statistically significant associations between nCRT and any of the primary outcome measures compared with nCT (Table 5).

### Aborted or Dose-Reduced Neoadjuvant Treatment versus Full Intended Treatment

Rates of major complications were similar between groups (43.8% A/R versus 42.6% full). The reoperation rate was 21.9% in the A/R group and 18.4% in patients undergoing full treatment; 90-day mortality was 5.2% in the A/R group and 3.9% in the full treatment group. Lengths of ICU and hospital stays were similar (Table 4).

**Table 4:** The 90-day outcomes of patients having aborted or dose-reduced neoadjuvant treatment or full intended neoadjuvant treatment prior to esophagectomy for esophageal cancer

	A/R	Full
	*n* (%)	*n* (%)
Patients	96 (100.0)	305 (100.0)
Complications defined by ECCG		
*Pulmonary*	*41* (*42.7*)	*122* (*40.0*)
Pneumonia	25 (26.0)	62 (20.3)
Pleural effusion requiring additional drainage procedure	18 (18.8)	70 (23.0)
Pneumothorax requiring treatment	1 (1.0)	11 (3.6)
Atelectasis mucous plugging requiring bronchoscopy	10 (10.4)	22 (7.2)
Respiratory failure requiring reintubation	10 (10.4)	18 (5.9)
Acute aspiration	5 (5.2)	11 (3.6)
Acute respiratory distress syndrome	2 (2.1)	11 (3.6)
Tracheobronchial injury	1 (1.0)	1 (0.3)
Chest tube maintenance for air leak for > 10 days postoperatively	1 (1.0)	2 (0.7)
*Cardiac*	*13* (*13.5*)	*51 *(*16.7*)
Cardiac arrest requiring CPR	2 (2.1)	0 (0.0)
Myocardial infarction	0 (0.0)	0 (0.0)
Dysrhythmia atrial requiring treatment	12 (12.5)	47 (15.4)
Dysrhythmia ventricular requiring treatment	0 (0.0)	1 (0.3)
Congestive heart failure requiring treatment	0 (0.0)	6 (2.0)
Pericarditis requiring treatment	0 (0.0)	0 (0.0)
*Gastrointestinal*	*21* (*21.9*)	*73* (*23.9*)
Anastomotic leak	11 (11.5)	34 (11.1)
Type 1	1 (1.0)	4 (1.3)
Type 2	7 (7.3)	18 (5.9)
Type 3	3 (3.1)	12 (3.9)
Conduit necrosis	1 (1.0)	14 (4.6)
Type 1	1 (1.0)	4 (1.3)
Type 2	0 (0.0)	1 (0.3)
Type 3	0 (0.0)	9 (3.0)
Ileus defined as small bowel dysfunction preventing or delaying enteral feeding	2 (2.1)	6 (2.0)
Small bowel obstruction	0 (0.0)	2 (0.7)
Feeding J-tube complication	3 (3.1)	7 (2.3)
Pyloromyotomy/pyloroplasty complication	1 (1.0)	3 (1.0)
*Clostridium difficile* infection	0 (0.0)	4 (1.3)
Gastrointestinal bleeding requiring intervention or transfusion	1 (1.0)	4 (1.3)
Delayed conduit emptying requiring intervention or delaying discharge or requiring maintenance of NG drainage > 7 days postoperatively	1 (1.0)	4 (1.3)
Pancreatitis	0 (0.0)	1 (0.3)
Pancreatic fistula	0 (0.0)	2 (0.7)
Liver dysfunction	0 (0.0)	0 (0.0)
Biliary leakage	0 (0.0)	2 (0.7)
*Urologic*	*3* (*3.1*)	*11* (*3.6*)
Acute renal insufficiency (defined as doubling of baseline creatinine)	1 (1.0)	10 (3.3)
Acute renal failure requiring dialysis	2 (2.1)	0 (0.0)
Urinary tract infection	1 (1.0)	2 (0.7)
Urinary retention requiring reinsertion of urinary catheter, delaying discharge, or discharge with urinary catheter	0 (0.0)	0 (0.0)
*Thromboembolic*	*2* (*2.1*)	*13* (*4.3*)
Deep venous thrombosis	0 (0.0)	1 (0.3)
Pulmonary embolus	2 (2.1)	11 (3.6)
Stroke (CVA)	0 (0.0)	1 (0.3)
Peripheral thrombophlebitis	0 (0.0)	1 (0.3)
*Neurologic*	*14* (*14.6*)	*32* (*10.5*)
Recurrent nerve injury	4 (4.2)	14 (4.6)
Type 1	3 (3.1)	13 (4.3)
Type 2	0 (0.0)	1 (0.3)
Type 3	0 (0.0)	0 (0.0)
Other neurologic injury	1 (1.0)	4 (1.3)
Acute delirium	9 (9.4)	16 (5.2)
Delirium due to alcohol withdrawal	1 (1.0)	0 (0.0)
*Infectious*	*14* (*14.6*)	*51* (*16.7*)
Wound infection requiring opening wound or antibiotics	1 (1.0)	12 (3.9)
Central IV-line infection requiring removal or antibiotics	0 (0.0)	5 (1.6)
Intraabdominal abscess	2 (2.1)	5 (1.6)
Intrathoracic abscess	3 (3.1)	21 (6.9)
Sepsis defined as positive blood culture	2 (2.1)	13 (4.3)
Other infections requiring antibiotics	7 (7.3)	14 (4.6)
*Wound/diaphragm*	*1* (*1.0*)	*12* (*3.9*)
Thoracic wound dehiscence	0 (0.0)	3 (1.0)
Abdominal wall wound dehiscence	1 (1.0)	9 (3.0)
Acute abdominal wall hernia	0 (0.0)	0 (0.0)
Acute diaphragmatic hernia	0 (0.0)	0 (0.0)
*Other*	*18* (*18.8*)	*55* (*18.0*)
Chyle leak		
Type 1	1 (1.0)	1 (0.3)
Type 2	3 (3.1)	13 (4.3)
Type 3	3 (3.1)	6 (2.0)
Reoperation for other reasons than bleeding, anastomotic leak, or conduit necrosis	12 (12.5)	38 (12.5)
Multiple organ dysfunction syndrome	0 (0.0)	3 (1.0)
Major complications	42 (43.8)	130 (42.6)
Reoperations	21 (21.9)	56 (18.4)
ICU stay (IQR)	2 (1–4)	2 (1–4)
Hospital stay (IQR)	15 (12–24)	14 (12–19.75)
90-day mortality	5 (5.2)	12 (3.9)

*A/R* Aborted or dose-reduced neoadjuvant treatment, *full* Full neoadjuvant treatment, *ECCG* Esophagectomy Complications Consensus Group, *CPR* Cardiopulmonary resuscitation, *NG* Nasogastric, *CVA* Cerebrovascular accident, *IV* Intravenous, *ICU* Intensive care unit, *IQR* Interquartile range

Pneumonia was more common in the A/R group (26.0% A/R versus 20.3% full). Rates of anastomotic leakage were similar (11.5% A/R versus 11.1% full), while conduit necrosis was more common in the full treatment group (1.0% A/R versus 4.6% full) (Table 4).

In complication categories, neurologic complications were slightly more common in the A/R group mainly due to a higher occurrence of delirium, while rates of other complication categories were roughly similar. (Table 4).

In multivariable analysis, aborted or dose-reduced neoadjuvant treatment was not associated with any adverse outcomes compared with full intended neoadjuvant treatment (Table 5).

**Table 5:** Logistic and Cox regression analyses of 90-day outcomes in patients undergoing neoadjuvant treatment compared with upfront surgery, neoadjuvant chemoradiotherapy compared with neoadjuvant chemotherapy, and in patients with aborted or dose-reduced neoadjuvant treatment compared with patients undergoing full intended neoadjuvant treatment

	nT	US	nCRT	nCT	A/R	Full
	*n* = 423	*n* = 351	*n* = 171	*n* = 249	*n* = 96	*n* = 305
	OR (95% CI)		OR (95% CI)		OR (95% CI)	
Individual complications						
Any complication						
Crude	**0.72 (0.53-0.97)**	1.00 (ref.)	1.13 (0.76–1.69)	1.00 (ref.)	0.82 (0.52–1.32)	1.00 (ref.)
Adjusted*	0.86 (0.61–1.21)	1.00 (ref.)	0.66 (0.39–1.11)	1.00 (ref.)	0.90 (0.54–1.49)	1.00 (ref.)
Major complication						
Crude	0.78 (0.58–1.03)	1.00 (ref.)	1.19 (0.80–1.77)	1.00 (ref.)	1.05 (0.66–1.66)	1.00 (ref.)
Adjusted*	0.87 (0.63–1.20)	1.00 (ref.)	0.75 (0.45–1.24)	1.00 (ref.)	1.09 (0.67–1.78)	1.00 (ref.)
Pneumonia						
Crude	0.98 (0.69–1.38)	1.00 (ref.)	**1.82 (1.14–2.91)**	1.00 (ref.)	1.38 (0.81–2.36)	1.00 (ref.)
Adjusted*	0.94 (0.64–1.40)	1.00 (ref.)	1.23 (0.68–2.23)	1.00 (ref.)	1.57 (0.88–2.78)	1.00 (ref.)
Anastomotic leak						
Crude	0.99 (0.65–1.50)	1.00 (ref.)	1.20 (0.68–2.12)	1.00 (ref.)	0.92 (0.46–1.83)	1.00 (ref.)
Adjusted*	0.84 (0.52–1.35)	1.00 (ref.)	0.93 (0.44–1.99)	1.00 (ref.)	0.89 (0.43–1.85)	1.00 (ref.)
Complication categories						
Pulmonary						
Crude	0.84 (0.64–1.12)	1.00 (ref.)	1.24 (0.83–1.84)	1.00 (ref.)	1.12 (0.70–1.78)	1.00 (ref.)
Adjusted*	0.89 (0.65–1.24)	1.00 (ref.)	0.77 (0.46–1.29)	1.00 (ref.)	1.37 (0.84–2.25)	1.00 (ref.)
Cardiac						
Crude	0.94 (0.65–1.37)	1.00 (ref.)	1.19 (0.71–2.00)	1.00 (ref.)	0.78 (0.40–1.51)	1.00 (ref.)
Adjusted*	0.95 (0.62–1.46)	1.00 (ref.)	0.62 (0.30–1.25)	1.00 (ref.)	0.77 (0.38–1.58)	1.00 (ref.)
Gastrointestinal						
Crude	1.11 (0.79–1.56)	1.00 (ref.)	1.21 (0.77–1.92)	1.00 (ref.)	0.89 (0.51–1.54)	1.00 (ref.)
Adjusted*	0.94 (0.64–1.39)	1.00 (ref.)	1.17 (0.65–2.11)	1.00 (ref.)	0.91 (0.51–1.63)	1.00 (ref.)
Urologic						
Crude	1.06 (0.47–2.36)	1.00 (ref.)	0.23 (0.05–1.06)	1.00 (ref.)	0.86 (0.24–3.16)	1.00 (ref.)
Adjusted*	1.38 (0.55–3.43)	1.00 (ref.)	0.28 (0.05–1.59)	1.00 (ref.)	1.25 (0.30–5.27)	1.00 (ref.)
Thromboembolic						
Crude	0.95 (0.46–1.97)	1.00 (ref.)	0.47 (0.15–1.49)	1.00 (ref.)	0.48 (0.11–2.16)	1.00 (ref.)
Adjusted*	1.14 (0.50–2.62)	1.00 (ref.)	0.63 (0.17–2.40)	1.00 (ref.)	0.48 (0.10–2.22)	1.00 (ref.)
Neurological						
Crude	0.70 (0.46–1.07)	1.00 (ref.)	**1.86 (1.00–3.44)**	1.00 (ref.)	1.46 (0.74–2.86)	1.00 (ref.)
Adjusted*	0.87 (0.54–1.41)	1.00 (ref.)	1.15 (0.52–2.56)	1.00 (ref.)	1.80 (0.87–3.74)	1.00 (ref.)
Infectious						
Crude	0.77 (0.53–1.12)	1.00 (ref.)	0.97 (0.56–1.66)	1.00 (ref.)	0.85 (0.45–1.62)	1.00 (ref.)
Adjusted*	0.83 (0.54–1.27)	1.00 (ref.)	0.76 (0.39–1.49)	1.00 (ref.)	0.95 (0.49–1.85)	1.00 (ref.)
Wound/diaphragm						
Crude	0.90 (0.40–1.99)	1.00 (ref.)	1.26 (0.42–3.81)	1.00 (ref.)	0.26 (0.03–2.00)	1.00 (ref.)
Adjusted*	1.03 (0.41–2.60)	1.00 (ref.)	0.72 (0.16–3.17)	1.00 (ref.)	0.28 (0.03–2.30)	1.00 (ref.)
Other						
Crude	1.07 (0.74–1.56)	1.00 (ref.)	1.30 (0.78–2.15)	1.00 (ref.)	1.05 (0.58–1.89)	1.00 (ref.)
Adjusted*	1.22 (0.79–1.88)	1.00 (ref.)	0.84 (0.44–1.61)	1.00 (ref.)	1.14 (0.60–2.15)	1.00 (ref.)
	**HR (95% CI)**		**HR (95% CI)**		**HR (95% CI)**	
	*n* = 423	*n* = 351	*n* = 171	*n* = 249	*n* = 96	*n* = 305
90-day mortality						
Crude	0.60 (0.32–1.13)	1.00 (ref.)	0.61 (0.21–1.72)	1.00 (ref.)	1.32 (0.47–3.75)	1.00 (ref.)
Adjusted*	0.68 (0.33–1.40)	1.00 (ref.)	0.44 (0.12–1.70)	1.00 (ref.)	1.57 (0.52–4.74)	1.00 (ref.)

^*^Adjusted for year of surgery, age, sex, tumor stage, comorbidity, histology, and surgical strategy.

Statistical significance indicated by bold font.

*nT* Neoadjuvant treatment, *US* Upfront surgery, *nCRT* Neoadjuvant chemoradiotherapy, *nCT* Neoadjuvant chemotherapy, *A/R* Aborted or dose-reduced neoadjuvant treatment, *full* Full neoadjuvant treatment, *OR* Odds ratio, *CI* Confidence interval, *HR* Hazard ratio, *ref*. Reference

## Discussion

The present study reports similar rates of overall postoperative complications, major complications, pneumonia, anastomotic leakage, and 90-day mortality in patients undergoing neoadjuvant treatment compared with those undergoing upfront surgery. These outcomes were also similar in patients undergoing neoadjuvant chemoradiotherapy compared with those undergoing neoadjuvant chemotherapy. Patients with aborted or dose-reduced neoadjuvant treatment had similar outcomes to patients undergoing full intended neoadjuvant treatment.

The landmark OE02, MAGIC, and CROSS trials, revolutionizing esophageal cancer management, did not report increased postoperative complications or mortality after neoadjuvant chemo(radio)therapy compared with surgery alone.^3,4,20^ However, these studies did not report complications according to ECCG or similar comprehensive reporting framework. Only one nationwide report (a Swedish study)^12^ has since been published, and experiences from actual clinical practice outside of strict clinical trial frameworks are therefore scarce. A meta-analysis of nine cohort studies found no differences in perioperative complications or mortality, however, the authors stated poor reporting on complications and a high risk of bias in several of the studies included.^21^ A meta-analysis on 12 trials found similar results, however, the generalizability to population level is questionable with trial-data only.^22^ Several studies comparing nCRT with surgery alone, however, have reported increased short-term mortality and cardiopulmonary morbidity, suggesting radiation adding to operative risk. A recent meta-analysis of 17 trials found a significant increase in postoperative morbidity but not mortality in patients undergoing nCRT.^8^ The aforementioned nationwide study from Sweden suggested an increased risk of complications after both nCT and nCRT compared with surgery alone, and higher postoperative mortality after nCRT compared with surgery alone.^12^ In the present study, there were some differences in the incidences of major complications and some of the complication categories, which were attenuated after adjusting for confounding, and nT was not associated with any adverse outcomes compared with US. These results are contrary to the Swedish nationwide results, albeit the Swedish study assessed nCRT and nCT separately. According to the authors, nCT mostly followed the MAGIC protocol in Sweden at the time of the study, however, they did not have detailed neoadjuvant treatment information available. In our study, nCT protocols were mostly EOX-derivatives.^23^ Potential differences in the heterogeneity of treatment protocols might explain differences in the outcomes of these studies. Other explaining factors could be differences in treatment indications between the countries in the early implementation of neoadjuvant treatment, invisible to analyses.

A clinical equipoise continuing for years has been the preferable approach to perioperative oncological treatment: nCT or nCRT? Current treatment guidelines do not recommend one over the other for esophageal adenocarcinoma.^2,9^ A few trials have investigated the matter. The Neo-AEGIS trial found no differences in mortality or major morbidity in patients with adenocarcinoma, suggesting continued clinical equipoise.^11^ The NeoRes trial, investigating both adenocarcinoma and squamous cell carcinoma, conversely found a higher comprehensive complications index and a higher 1-year mortality in nCRT compared with nCT.^10^ The recent ESOPEC trial comparing perioperative 5-FU, leucovorin, oxaliplatin, and docetaxel (FLOT) with CROSS for adenocarcinoma reported an increase in 90-day mortality in patients undergoing CROSS (3.1% versus 5.6%), with no difference in complications.^24^ An international study by the OGAA comparing FLOT and CROSS had similar results prior to the ESOPEC trial, with no difference in overall complications, but a significant increase in 90-day mortality with CROSS, likely mediated by cardiopulmonary complications.^13^ A European propensity-matched analysis of patients from ten expert centers found no significant difference in complications apart from a higher rate of anastomotic leakage in patients undergoing nCRT compared with nCT, which most likely was due to difference in surgical technique not taken into account in the matching process.^25^ The Swedish nationwide study found no differences in outcomes after nCRT compared with nCT.^12^ In the current study, nCRT was not associated with any adverse outcomes compared with nCT. The initial difference in the rate of pneumonia, most likely resulting from a more frequent use of tMIO in the nCT group (Supplementary Table 1), was attenuated after adjusting for surgical approach.^26^ Similarly, the difference in neurological complications (mediated by the higher incidence of recurrent nerve injury with nCRT), was attenuated after adjusting for confounding. The difference in mortality outcomes of the current study compared with the ESOPEC trial and the study by OGAA might be explained by the fact that only 9% of patients in the current study received CROSS as it was introduced in 2012, and other patients in the nCRT group received varying doses of radiation. Dose-dependent decreases in diffusion capacity have been reported in some studies, possibly mediating an increased risk of postoperative morbidity.^27^ On the contrary, nCT protocols were less severe than FLOT in the current study.

Patients having neoadjuvant treatment complications might be treated as “high-risk” patients in surgery. In the present study, rates of major complications were similar between the A/R and full treatment groups, while pneumonia was slightly more common in the A/R group; 90-day mortality was slightly higher in the A/R group. However, in multivariable analysis, these differences were attenuated. These results suggest that patients having neoadjuvant treatment complications leading to aborting treatment or a dose-reduction might not be at greater operative risk, and most importantly, that surgery should not be withheld solely due to neoadjuvant treatment complications in otherwise eligible patients. This analysis was somewhat limited since patients with neoadjuvant treatment complications who did not ultimately undergo surgery were not available.

The strengths of this study include the population-based, nationwide design and an unselected cohort, reducing selection bias and providing a real-world setting for analyses lacking from most previous studies. Sample sizes were large enough for adequate statistical power. Another strength is the comprehensive assessment of complications and adherence to a standardized framework in recording of outcomes, allowing adequate comparison with future studies. The main limitation of this study is its retrospective design. Even though patient records were meticulously assessed by experts, complication data could have been missed or never documented. Patient selection for treatment modality in the early implementation of neoadjuvant treatment was not random. Even though patient characteristics were largely similar between groups and adjustments for confounding were made, some bias could remain in the analyses. The relatively long study period could introduce era effects, as treatment outcomes have improved following an overall secular trend. This was taken into account by adjusting for the year of surgery. Information on perioperative care and the use of Enhanced Recovery After Surgery (ERAS) protocols were not available and could not be accounted for in the analyses. Another limitation is the heterogeneity in the neoadjuvant treatment protocols used during the study period. As a majority of the patients received EOX-derived neoadjuvant therapies, the results of the current study might not be applicable to those receiving FLOT, which has replaced these therapies in current practice for patients able to tolerate it. The results are, however, still relevant, as a significant portion of patients are not able to tolerate a full FLOT-protocol, and EOX-derived therapies are still in wide use.

To conclude, this nationwide, population-based study reports no increase in postoperative morbidity or mortality in patients undergoing neoadjuvant treatment compared with those undergoing upfront surgery, nor in patients undergoing nCRT compared with nCT. These results suggest that the choice of pre- or perioperative oncological treatment could be made with long-term survival outcomes in mind without said choice affecting surgical risk. Patients having neoadjuvant treatment complications were not at greater risk of surgical complications compared with patients undergoing full dose neoadjuvant treatment, suggesting that these patients should not be withheld from surgery if otherwise eligible. Future studies should monitor the use of FLOT in nationwide practice in terms of postoperative morbidity and mortality.

## Supplementary Information

Below is the link to the electronic supplementary material.

Supplementary file1 (DOCX 186 KB)

## References

[1] Sung H, Ferlay J, Siegel RL, et al. Global cancer statistics 2020: GLOBOCAN estimates of incidence and mortality worldwide for 36 cancers in 185 countries. *CA Cancer J Clin*. 2021. 10.3322/caac.21660. 33538338 10.3322/caac.21660

[2] Obermannová R, Alsina M, Cervantes A, et al. Oesophageal cancer: ESMO Clinical Practice Guideline for diagnosis, treatment and follow-up. *Ann Oncol*. 2022. 10.1016/j.annonc.2022.07.003. 35914638 10.1016/j.annonc.2022.07.003

[3] Cunningham D, Allum WH, Stenning SP, et al. Perioperative chemotherapy versus surgery alone for resectable gastroesophageal cancer. *N Engl J Med*. 2006. 10.1056/nejmoa055531. 16822992 10.1056/NEJMoa055531

[4] Allum WH, Stenning SP, Bancewicz J, et al. Long-term results of a randomized trial of surgery with or without preoperative chemotherapy in esophageal cancer. *J Clin Oncol*. 2009. 10.1200/JCO.2009.22.2083. 19770374 10.1200/JCO.2009.22.2083

[5] Eyck BM, Van Lanschot JJB, Hulshof MCCM, et al. Ten-year outcome of neoadjuvant chemoradiotherapy plus surgery for esophageal cancer: the randomized controlled CROSS Trial. *J Clin Oncol*. 2021. 10.1200/JCO.20.03614. 33891478 10.1200/JCO.20.03614

[6] Mariette C, Dahan L, Mornex F, et al. Surgery alone versus chemoradiotherapy followed by surgery for stage I and II esophageal cancer: final analysis of randomized controlled phase III trial FFCD 9901. *J Clin Oncol*. 2014. 10.1200/JCO.2013.53.6532. 24982463 10.1200/JCO.2013.53.6532

[7] Bosset JF, Gignoux M, Triboulet JP, et al. Chemoradiotherapy followed by surgery compared with surgery alone in squamous-cell cancer of the esophagus. *N Eng J Med*. 1997. 10.1056/nejm199707173370304. 10.1056/NEJM199707173370304 9219702

[8] Ronellenfitsch U. Preoperative chemoradiotherapy vs. chemotherapy for adenocarcinoma of the esophagus and esophagogastric junction (AEG): systematic review with individual patient data (IPD) network meta-analysis (NMA). *Eur J Surg Oncol*. 2024. 10.1016/j.ejso.2023.107360.

[9] Ajani JA, D’Amico TA, Bentrem DJ, et al. Esophageal and esophagogastric junction cancers, version 2.2019, NCCN Clinical Practice Guidelines in Oncology. *J Natl Compr Cancer Netw*. 2019. 10.6004/jnccn.2019.0033. 10.6004/jnccn.2019.0033 31319389

[10] Klevebro F, von Döbeln GA, Wang N, et al. A randomized clinical trial of neoadjuvant chemotherapy versus neoadjuvant chemoradiotherapy for cancer of the oesophagus or gastro-oesophageal junction. *Ann Oncol*. 2016. 10.1093/annonc/mdw010. 26782957 10.1093/annonc/mdw010

[11] Reynolds JV, Preston SR, O’Neill B, et al. Trimodality therapy versus perioperative chemotherapy in the management of locally advanced adenocarcinoma of the oesophagus and oesophagogastric junction (Neo-AEGIS): an open-label, randomised, phase 3 trial. *Lancet Gastroenterol Hepatol*. 2023. 10.1016/S2468-1253(23)00243-1. 37734399 10.1016/S2468-1253(23)00243-1 PMC10567579

[12] Klevebro F, Lindblad M, Johansson J, et al. Outcome of neoadjuvant therapies for cancer of the oesophagus or gastro-oesophageal junction based on a national data registry. *Br J Surg*. 2016. 10.1002/bjs.10304. 27689845 10.1002/bjs.10304

[13] Alderson D, Bundred J, Steering Committee, et al. Postoperative and pathological outcomes of CROSS and FLOT as neoadjuvant therapy for esophageal and junctional adenocarcinoma: an international cohort study from the Oesophagogastric Anastomosis Audit (OGAA). *Ann Surg*. 2023. 10.1097/SLA.0000000000005394. 10.1097/SLA.0000000000005394 35099168

[14] Kauppila JH, Ohtonen P, Karttunen TJ, et al. Finnish National Esophago-Gastric Cancer Cohort (FINEGO) for studying outcomes after oesophageal and gastric cancer surgery: a protocol for a retrospective, population-based, nationwide cohort study in Finland. *BMJ Open*. 2019. 10.1136/bmjopen-2018-024094. 30782726 10.1136/bmjopen-2018-024094 PMC6340442

[15] Sund R. Quality of the Finnish Hospital Discharge Register: a systematic review. *Scand J Public Health*. 2012;40(6):505–15. 22899561 10.1177/1403494812456637

[16] Leinonen MK, Miettinen J, Heikkinen S, et al. Quality measures of the population-based Finnish Cancer Registry indicate sound data quality for solid malignant tumours. *Eur J Cancer*. 2017;77:31–9. 28350996 10.1016/j.ejca.2017.02.017

[17] Kauppila JH. Completeness of esophageal cancer diagnosis in the Finnish Cancer Registry and hospital discharge registry, a nationwide study in Finland. *Acta Oncol (Madr)*. 2020;59:1329–32. 10.1080/0284186X.2020.1792547 32684064

[18] Low DE, Alderson D, Cecconello I, et al. International consensus on standardization of data collection for complications associated with esophagectomy: Esophagectomy Complications Consensus Group (ECCG). *Ann Surg*. 2015. 10.1097/SLA.0000000000001098. 25607756 10.1097/SLA.0000000000001098

[19] Dindo D, Demartines N, Clavien PA. Classification of surgical complications: a new proposal with evaluation in a cohort of 6336 patients and results of a survey. *Ann Surg*. 2004. 10.1097/01.sla.0000133083.54934.ae. 15273542 10.1097/01.sla.0000133083.54934.ae PMC1360123

[20] van Hagen P, Hulshof MCCM, van Lanschot JJB, et al. Preoperative chemoradiotherapy for esophageal or junctional cancer. *N Eng J Med*. 2012. 10.1056/nejmoa1112088. 10.1056/NEJMoa1112088 22646630

[21] Kidane B, Korst RJ, Weksler B, et al. Neoadjuvant therapy vs upfront surgery for clinical T2N0 esophageal cancer: a systematic review. *Ann Thorac Surg*. 2019. 10.1016/j.athoracsur.2019.04.008. 31077657 10.1016/j.athoracsur.2019.04.008

[22] Faron M, Cheugoua-Zanetsie AM, Thirion P, et al. Individual patient data meta-analysis of neoadjuvant chemotherapy followed by surgery versus upfront surgery for carcinoma of the oesophagus or the gastro-oesophageal junction. *Eur J Cancer*. 2021. 10.1016/j.ejca.2021.08.014. 34555647 10.1016/j.ejca.2021.08.014

[23] Cunningham D, Starling N, Rao S, et al. Capecitabine and oxaliplatin for advanced esophagogastric cancer. *N Eng J Med*. 2008. 10.1056/nejmoa073149. 10.1056/NEJMoa073149 18172173

[24] Hoeppner J, Brunner T, Schmoor C, et al. Perioperative chemotherapy or preoperative chemoradiotherapy in esophageal cancer. *N Eng J Med*. 2025. 10.1056/NEJMoa2409408. 10.1056/NEJMoa2409408 39842010

[25] Markar SR, Noordman BJ, Mackenzie H, et al. Multimodality treatment for esophageal adenocarcinoma: multi-center propensity-score matched study. *Ann Oncol*. 2017. 10.1093/annonc/mdw560. 28039180 10.1093/annonc/mdw560 PMC5391716

[26] Biere SSAY, Van Berge Henegouwen MI, Maas KW, et al. Minimally invasive versus open oesophagectomy for patients with oesophageal cancer: a multicentre, open-label, randomised controlled trial. *Lancet*. 2012. 10.1016/S0140-6736(12)60516-9. 22552194 10.1016/S0140-6736(12)60516-9

[27] Gergel TJ, Leichman L, Nava HR, et al. Effect of concurrent radiation therapy and chemotherapy on pulmonary function in patients with esophageal cancer: dose-volume histogram analysis. *Cancer J*. 2002. 10.1097/00130404-200211000-00009. 12500854 10.1097/00130404-200211000-00009

