# Supplementary material for: Neoadjuvant Treatment and Postoperative Complications After Surgery for Esophageal Cancer: A Population-Based, Nationwide Study in Finland
Source: Ann Surg Oncol. 2025 Aug 6;32(10):7914–28. doi: 10.1245/s10434-025-17945-y (PMC12454505; doi:10.1245/s10434-025-17945-y)
Supplement: Supplementary file 1 — Supplementary file1 (DOCX 186 KB) [file 10434_2025_17945_MOESM1_ESM.docx]

**Supplemental table 1.** Characteristics of patients undergoing neoadjuvant chemoradiotherapy or neoadjuvant chemotherapy in Finland in 2005-2016.

|  | **nCRT** | **nCT** |  |
| --- | --- | --- | --- |
|  | **n (%)** | **n (%)** | **p-value** |
| **Total** | 171 (100.0) | 249 (100.0) |  |
| **Sex** |  |  | **0.044** |
| Male | 127 (74.3) | 206 (82.7) |  |
| Female | 44 (25.7) | 43 (17.3) |  |
| **Age** |  |  | 0.459 |
| Median (IQR) | 63 (58-70) | 63 (57-69) |  |
| Clinical stage |  |  | **<0.001** |
| 0 | 0 (0.0) | 0 (0.0) |  |
| I | 1 (0.6) | 0 (0.0) |  |
| II | 35 (20.5) | 11 (4.4) |  |
| III | 107 (62.6) | 188 (75.2) |  |
| IV | 28 (16.4) | 51 (20.4) |  |
| **Pathological stage** |  |  | **<0.001** |
| 0 | 31 (18.6) | 6 (2.4) |  |
| I | 36 (21.6) | 50 (20.2) |  |
| II | 32 (19.2) | 44 (17.8) |  |
| III | 55 (32.9) | 114 (46.2) |  |
| IV | 13 (7.8) | 33 (13.4) |  |
| **CCI** |  |  | 0.312 |
| 0 | 88 (51.5) | 144 (57.8) |  |
| 1 | 60 (35.1) | 67 (26.9) |  |
| 2 | 16 (9.4) | 24 (9.6) |  |
| ≥3 | 14 (5.6) | 14 (5.6) |  |
| **Histology** |  |  | **<0.001** |
| Adenocarcinoma | 82 (48.0) | 238 (95.6) |  |
| Squamous cell carcinoma | 89 (52.0) | 11 (4.4) |  |
| **Tumor location** |  |  | **<0.001** |
| Upper | 6 (3.5) | 2 (0.8) |  |
| Middle | 49 (28.7) | 6 (2.4) |  |
| Lower | 93 (54.4) | 113 (45.4) |  |
| GO-junction | 23 (13.5) | 128 (51.4) |  |
| **Mode of surgery** |  |  | **0.001** |
| Open | 108 (63.2) | 114 (45.8) |  |
| Hybrid | 15 (8.8) | 25 (10.0) |  |
| tMIO | 48 (28.1) | 110 (44.2) |  |
| **Type of resection** |  |  | **<0.001** |
| Ivor-Lewis | 94 (55.0) | 223 (89.6) |  |
| McKeown | 70 (40.9) | 9 (3.6) |  |
| Transhiatal | 5 (2.9) | 9 (3.6) |  |
| Left thoracoabdominal | 1 (0.6) | 0 (0.0) |  |
| Proximal gastrectomy | 0 (0.0) | 1 (0.4) |  |
| Combined gastro-oesophagectomy | 1 (0.6) | 7 (2.8) |  |
| **Conduit material** |  |  | 0.397 |
| Stomach | 168 (98.2) | 242 (97.2) |  |
| Small intestine | 1 (0.6) | 2 (0.8) |  |
| Colon | 1 (0.6) | 5 (2.0) |  |
| Unclear or other | 1 (0.6) | 0 (0.0) |  |
| **Location of anastomosis** |  |  | **<0.001** |
| Neck | 75 (43.9) | 14 (5.6) |  |
| Thorax | 95 (55.6) | 235 (94.4) |  |
| Unclear or no anastomosis | 1 (0.6) | 0 (0.0) |  |
| **Resection radicality** |  |  | 0.360 |
| R0 | 154 (91.1) | 227 (91.2) |  |
| R1 | 10 (5.9) | 19 (7.6) |  |
| R2 | 5 (3.0) | 3 (1.2) |  |
|  |  |  |  |
| **Lymph node yield** |  |  | **0.001** |
| 0-16 | 86 (51.8) | 86 (34.5) |  |
| ≥17 | 80 (48.2) | 163 (65.5) |  |

Abbreviations: nCRT, neoadjuvant chemoradiotherapy; nCT, neoadjuvant chemotherapy; IQR, interquartile range; CCI, Charlson comorbidity index; tMIO, totally minimally invasive oesophagectomy

Statistical significance indicated by **bold font.**
